# Interventional cytopathology and cancer in Peru: how to act during COVID-19?

**DOI:** 10.3332/ecancer.2020.1152

**Published:** 2020-12-07

**Authors:** Milagros Abad-Licham, Juan Astigueta, Caddie Laberiano Fernández, Himelda Chávez Torres, Grisnery Maquera Torres, Edwin Figueroa, Ricardo Bardales

**Affiliations:** 1 School of Medicine, Antenor Orrego Private University, Trujillo 13007, Peru; 2 Pathological Oncology Department, Northern Regional Institute of Neoplastic Diseases, Trujillo 13600, Peru; 3 Centre of Excellence in Pathological Oncology, Trujillo 13007, Peru; 4 Department of Uro-Oncology, Northern Regional Institute of Neoplastic Diseases, Trujillo 13600, Peru; 5 Arias Stella Institute of Pathology and Molecular Biology, Lima 15000, Peru; 6 Peruvian University of Applied Sciences, Lima 15000, Peru; 7 Cytology service, Edgardo Rebagliati Martins National Hospital, Lima 15000, Peru; 8 National University of San Marcos, Lima 15000, Peru; 9 Functional Cytopathology Unit, National Institute of Neoplastic Diseases, Lima 15000, Peru; 10 Head and Neck Department, Northern Regional Institute of Neoplastic Diseases, Trujillo 13600, Peru; 11 Precision Pathology, CA 95826, USA; a https://orcid.org/0000-0002-3530-6937; b https://orcid.org/0000-0001-5984-3270; c https://orcid.org/0000-0003-4513-6123; d https://orcid.org/0000-0003-4519-4745; e https://orcid.org/0000-0001-6203-3068

**Keywords:** fine-needle biopsy, cancer, COVID-19, biosafety

## Abstract

The worldwide health crisis due to SARS-CoV-2 (severe acute respiratory syndrome coronavirus 2) has affected all healthcare systems. Low- and middle-income countries have needed to establish health strategies to combat the pandemic, many of which have collaterally affected the diagnosis and treatment of other illnesses. One of these other illnesses is cancer, which in Peru represents the primary cause of mortality. In recent decades, interventional cytopathology with fine-needle biopsy techniques has emerged as a minimally invasive, rapid, economical and effective procedure for diagnosing and staging cancer. However, in the current health context, it is confronted by the challenge of continuing to function in spite of the pandemic. This article reviews the existing literature on interventional cytopathology, the risk of infection from SARS-CoV-2 and biosafety and provides recommendations for carrying out said procedures for the benefit of the patient and the safety of healthcare staff.

## Introduction

Cancer is a serious public health problem worldwide, and, in Peru, represents the primary cause of mortality [1]. The associated disability caused by cancer and the high social cost constitutes a challenge for our debilitated healthcare system, which must also overcome geographic and cultural barriers, population growth and migration and the associated changes in the prevalence and distribution of risk factors, several of which are related to low socioeconomic levels [2]. In this context, Peru has established strategies to control the disease and has set as a goal the reduction of advanced cancer in its population [2–4]. This includes interventional cytopathology as an effective, simple and economical technique with minimal complications, though it requires adequate training in cytopathology and the necessary equipment [5, 6].

In spite of its virtues and the growing interest in interventional cytopathology evidenced in several national publications [6–9], today’s cytopathology laboratory faces a great challenge: functioning during the pandemic caused by SARS-CoV-2, which has brought down healthcare systems around the world [10]. Healthcare professionals have a high risk of infection due to contact with infected people with or without symptoms, limited availability of personal protective equipment (PPE), inadequate healthcare service infrastructure to confront infectious outbreaks of this magnitude and the precariousness the foregoing in low- and middle-income countries. In this context, laboratory staff, including interventional cytopathologists, are in a high-risk group [10–13].

Conscious of the importance of continuing with diagnostic work, in this article, the recommendations made in the literature are analysed to guarantee correct functioning in the area of interventional cytopathology so that oncology cytodiagnosis is prioritised for the benefit of the patient, but also for the care and health of laboratory staff.

## Methodology

A literature review was carried out on PubMed, SCOPUS and LILACS, using the terms ‘fine needle biopsy’, ‘cancer’, ‘COVID-19’ and ‘biosafety’, in both English and Spanish. The bibliographic search was carried out by the authors, who, in virtual form, selected, analysed, discussed and wrote the recommendations.

## Results and discussion

### Interventional cytopathology

Interventional cytopathology is a branch of anatomical pathology in which the cytopathologist carries out minimally invasive procedures on palpable masses or masses identified by ultrasound, using a fine calibre needle (fine needle) to obtain the sample and issue a cytological diagnosis. There are two variants on the technique: fine needle aspiration (FNA) biopsy, and biopsy without aspiration, also known as the fine needle capillary (FNC) biopsy technique. Both have a high degree of diagnostic accuracy and in some cases are used together to complement each other, improving their validity [14, 15]. Interventional cytopathology traces its history to Arabic medicine, with previous contributions from Dudgeon and Patrick in the United Kingdom, Martin and Ellis in the United States and Zajdela, Zajicek and Franzen in the Netherlands [16]. In Peru, it started formally in the final decade of the last century with Columbie, Somocurcio and collaborators routinely using FNA to approach thyroid gland tumours [17]**.** The diffusion of this procedure to other hospitals in Lima and the provinces crept forward slowly. In the oncology space, in 2007, the National Institute of Neoplastic Diseases in Lima included the diagnostic protocol for head and neck tumours and subsequently for other neoplasms. Four years later, Bardales and Abad implemented FNA and FNC in the Northern Regional Institute of Neoplastic Diseases, using predominantly 27 and 25G needles. This experiment constituted the first national publication on this matter [7]. To date, interventional cytopathology has been adopted in other institutions for early diagnosis in oncology, staging, evaluation of the response to treatment and even as a screening test [5, 6].

FNA and FNC are techniques carried out with a fine needle which reduces the presence of haematic matter, which often makes diagnosis difficult. In both variations (FNA and FNC), the sample obtained remains in the needle, with the exception of the cysts, to then be ejected with the help of a syringe onto a microscope slide [14, 15, 18]. For the purposes of biosafety within the current pandemic, this last step holds the greatest risk due to the generation of aerosols and the possibility of infection [12]. Once the sample has been spread on the slides, they must immediately be fixed in 96% alcohol for subsequent and definitive staining. The sample obtained can also be used to prepare a cell block, immunocytochemistry, genetic study and flow cytometry, among others [13, 19].

This technique and its benefits are captured in different articles. It is simple, outpatient, rapid, safe, economical and has high diagnostic accuracy (sensitivity 80%–100% and specificity around 99%) [7, 8]. It is reproducible with minimal complications, with adequate staff training for those performing the procedure, processing the sample and interpreting the results being the most important condition for its success. An adequately trained cytopathologist can also carry out immediate assessments on the quality of samples obtained using rapid staining to avoid unsatisfactory results, saving time in the therapeutic decision-making process [14, 15, 18, 19].

The disadvantages of the technique are minimal; however, they must be considered. From the point of view of the procedure, although bleeding is rare, localised and easily controlled, it should be borne in mind, particularly in patients with clotting disorders. Communication with the clinic should be constant and fluid to improve the diagnostic accuracy of the interventional cytopathology [14, 18, 19].

### SARS-CoV-2

SARS-CoV-2 is responsible for the current pandemic, which in Peru, on the date of this writing, reports more than 525,000 cases and 26,000 deaths with a mortality rate of 4.96% [20, 21]. The pathophysiology, and clinical and therapeutic behaviour of COVID-19 are still not well established. However, the principle modes of infection have been identified, and this knowledge has been fundamental in designing adequate prevention and biosafety strategies [10, 22, 23]. The illness spreads through the respiratory secretions of an infected person by coughing, sneezing or speaking; it is known that the virus’s main point of entry is respiratory, followed by the oral and conjunctival mucosae [12, 13].

Regarding the source of the samples and their level of virulence, these have been classified as high and low risk. Sources considered high risk are those from the respiratory tract, such as sputum, nasopharyngeal swabs, broncho-alveolar lavages, pleural fluid, as well as conjunctival secretions and any specimen obtained with a fine needle, rubbing or coming from a suspected or confirmed COVID-19 patient [12, 24, 25]. The elimination time of the SARS-CoV-2 RNA in the samples of infected patients remains partially unknown, hence the importance of personal protection and correct processing of the specimen [12].

### Role of intervention in oncology decisions

Interventional cytopathology has an important role in cancer, not only in the diagnosis of patients with palpable and non-palpable tumours, but also in staging, evaluating recurrence, progression and monitoring of the disease, as well as the use of diagnostic cytology in planning a surgical procedure and the radicality of same. In summary, it is a fundamental pillar for making management decisions. It can also provide a definitive diagnosis, supported in the clinical context with imaging and auxiliary studies. Flow cytometry, immunocytochemistry, molecular analysis and genetic studies increase the diagnostic value of the technique [7, 15, 19].

The technique has demonstrated its virtues as summarised in Table 1. The previously known advantages of this minimally invasive procedure are reinforced in the context of the pandemic, such as, for example: speed in scheduling the procedure; low or minimal costs (no need for preparatory exams, surgery, instruments, special equipment or nursing staff); the patient usually easily agrees to the procedure when it is explained to them that it is fast, safe, largely painless, outpatient, minimally traumatic and essentially without complications. Furthermore, sample processing is rapid, and a second sample can be taken should the first prove insufficient [26].

In the current health context, the discussion focuses on risks the procedure poses to the cytopathologist. In cases of necessity, and when its utility and advantages outweigh the risks, it will have to be performed under optimal biosafety conditions [24]. The evidence shows that it reduces the number of unnecessary surgeries, in many cases eliminating the need for intraoperative biopsies, and shortens the waiting time for results, enabling earlier therapeutic interventions [15, 17, 19, 26]. During this pandemic, the importance of interventional cytopathology as a diagnostic procedure is reinforced.

### Laboratory staff

The arrival of COVID-19 in our country and the world has changed the manner in which we act and work. The potential presence of the virus in cytology samples has forced us to adopt strict biosafety measures, starting with the workers and their duties. Considering that the current situation requires the presence of the minimum necessary number of staff in a work area, interventional cytopathology procedures should include the cytopathologist responsible for taking the sample, and support staff charged with logistics, moving, staining and technical processes, all adequately trained [15, 24].

Staff should use full PPE which includes: N95 (or higher) mask, disposable gown with long sleeves, gloves (preferably nitrile), surgical cap, boots, face shield and safety glasses. Before and after the procedure, it is important to wash hands with soap and water. Correctly wearing and removing PPE is also important [11–13, 15–25] and should be carried out in an area designed for this purpose [23].

### Procedure and processing

Interventional cytopathology procedures are carried out in accordance with a plan and previous evaluation of the patient’s clinical history by the cytopathologist. To this end, it is important that the study request includes the relevant clinical data and justification for the study. The COVID-19 status of the patient must be specified, indicating whether they are positive, suspected or negative. In emergency or controversial cases, multidisciplinary evaluation is recommended to avoid unnecessary exposure [13].

The patients should wear a mask, preferably surgical, throughout the entire procedure [23], except in cases wherein the lesion is found in the oral cavity. They should be admitted alone; if this is not possible, a member of healthcare staff from the same establishment, who will be adequately protected, will accompany them. The procedure should be carried out in an environment specific to this purpose, ventilated and with adequate illumination [12, 23, 24]. We recommend a checklist of the materials used during the procedure, including signed informed consent.

**Figure 1.**
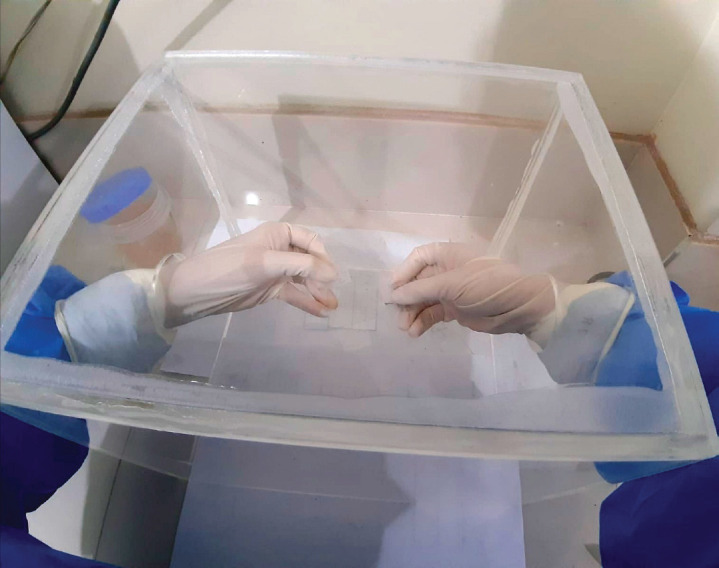
Acrylic isolation booth to reduce the possibility of contamination by aerosols at the time of expulsion and extension.

The chosen technique depends upon the operator. In the bibliographic review, we did not find any evidence about the level of protection, favouring FNA versus FNC, nor the number of samples to take (passes) or the number of slides to prepare. In our experience, we suggest minimising the number of passes (three or four), and designating one for use as a sample for the cell block. The critical point is in the expulsion of the sample, pointing the bevel of the needle downwards and in contact with the slide to minimise aeration of the sample [15]. Some authors recommend that, after piercing, the sample be transferred to the laboratory to be processed in a Class II biosafety cabinet [15, 25]. If this is not possible, an alternative is proposed by the Indian Academy of Cytologists, who suggest that expulsion of the material from the needle should be gentle and that the cytopathologist, wearing full PPE, should maintain the greatest distance possible whilst manipulating the sample [23], followed by immediate fixation in 96% alcohol and staining with Papanicolaou or hematoxylin eosin [22]. Another option is the use of a system of physical barriers during the expulsion and extension process (Figure 1). In this context, it is prudent to not carry out immediate evaluations, especially in the same puncture location [23, 24, 27]. The recommendation is to move the already fixed sample to the laboratory to colour the slide, assess the quality and give a preliminary assessment.

On finishing the procedure, the equipment and the surfaces that have been in contact with the sample and the patient must be cleaned and disinfected [28]. Afterwards, all disposable material, including the PPE, should be thrown out in a container with a bio-hazard disposal bag [29]. The reusable equipment, such as the safety glasses and visor, should be disinfected or thrown away according to the biosafety rules of each institution [28, 29]. In cases involving patients who have tested positive for COVID-19, the procedure should be scheduled for the end of the shift to minimise the risk of infection. Ideally, only a cell block should be performed, and the sample manipulated in a Class II biosafety cabinet [29]. The cytological report would be prepared in a conventional form and according to the international recommendations for each anatomical region.

### The challenge: to continue to function

The health strategies designed to combat this pandemic and the need to continue supporting patients with cancer has forced us to implement adequate biosafety measures for the cytology laboratory and the interventional area to continue functioning. The literature recommends continuing with interventions in necessary cases, such as those patients with oncological diseases and in which interventional cytology is fundamental for decision making [13, 22, 23, 24]. To that effect, and considering that currently the majority of fresh biological samples are potentially infected, the infection routes and high virulence of SARS-CoV-2, as well as its survival on surfaces is proven [12, 13, 27, 28], we should replace the premise of ‘correct and early diagnosis’, with ‘correct and opportune diagnosis’, prioritising the fixation of the sample in alcohol as a fundamental step to inactivate the virus and protect the laboratory staff.

Measures should include strict biosafety procedures which include personal protection using adequate equipment (PPE), frequent hand washing, correct hygiene practices, properly scheduled shift work and refreshment breaks should be minimal and staggered in order to avoiding overcrowding. To reduce contact between people, limiting to strictly necessary staff for both sample collection and processing, and they should keep a minimum social distance of 1.5 metres [24, 25, 27]. It is recommended that this distance also be applied to the pathology offices, trying wherever possible to have only one person per office. It is also important that all staff who participate in interventions are trained, and that they follow the applicable biosafety measures in each laboratory [12, 13, 27]. Table 2 gives a summary of the recommendations.

## Conclusions

Interventional cytopathology should continue as a functioning area, but with all the means necessary for the biosafety of the staff. This includes one adequate environment for the procedure and another for processing; use of complete PPE; equipment for the technique; trained staff and permanent communication with the treating doctor.

Any member of the interventional cytopathology team who has had extended contact with a patient having a suspected or confirmed SARS-CoV-2 infection, or has been potentially exposed to the virus during the procedure or preparation of the sample, must communicate this immediately to management and Occupational Health and/or Epidemiology to establish the procedure to follow in accordance with the norms of the health establishment. In cases of infection in the community, the protocols, which have already been established, will be followed.

The COVID-19 pandemic has demonstrated to the entire world, mainly to low- and middle-income countries, the shortages in medical practices, inadequate biosecurity protocols and the scarcity of materials and teams. These deficiencies should not limit continued care, particularly for patients with oncological diseases. The area of interventional cytopathology for all the aforementioned advantages must reinforce its role in the diagnostic process, adapting itself to the so-called ‘new normal’ for the benefit of the patients. It is also very important to maximise the biosafety protocols to protect the health of the healthcare workers, a fundamental mainstay for continued aid.

## Conflicts of interest

The authors have not declared any conflicts of interest.

## Funding

The authors state that this work is self-financed.

## Figures and Tables

**Table 1.** Interventional cytopathology: analysis of technical advantages in the context of the COVID-19 pandemic.

Patient acceptance of the procedure	Yes
Coagulation tests	Not essential
Need for surgical risk	No
Need for surgeon	No
Special materials and instruments	No
Recovery room	No
Number of staff needed	1 to 2
PPE	Complete
Speed of procedure	Minutes
Soft tissue trauma	Minimal
Pain	Minimal
Need for analgesics	Usually not needed
Risk of complications	Minimal
Sample processing time	Minutes
Waiting time for result	Less than 24 hours
Evaluation of sample quality	May be immediate
Repetition of procedure	Immediate
Costs	Low
Risk of infection for operators during the process	During the expulsion and extension of the sample

**Table 2.** Summary of recommendations for interventional cytopathology during COVID-19.

Healthcare professional older than 60 or with co-morbidities should avoid working. Scheduled shift work and/or staggered shifts. Conserve minimal social distancing of 1.5 m. The study request should include complete clinical information and indicate whether the patient is COVID-19 positive, suspected or negative. Positive or suspected COVID-19 patients should be scheduled at the end of the shift to avoid contaminating the work area.Wash hands with soap and water before and after the procedure. Staff should use full PPE: N95 or higher mask, long sleeved gown, gloves (preferably nitrile), surgical cap, boots, face shield and safety glasses.The procedure should be carried out in a ventilated environment, and whenever possible the patient should be admitted alone. The patient should wear a surgical mask throughout the entire procedure. Whenever possible, limit the number of samples obtained (one of them for the cell block). Expulsion of the material from the needle should be gentle, with the bevel pointing down and in contact with the slide, and with the greatest distance possible during preparation for extension.Carry out evaluations immediately on fresh samples only if it is necessary and fix the slide in 96% alcohol.On finishing, the equipment, physical environment and surfaces in contact with the sample and the patient must be cleaned and disinfected.The cytological report will be produced in a conventional form. Tell the appropriate person about any staff contact with a patient with suspected or confirmed SARS-CoV-2 infection, or if there have been any technical complications or problems with the PPE during the procedure.Ideally, it is recommended that the technical processing of the sample be performed in a Class II biosafety cabinet.

## References

[1] Ministerio de Salud. Centro Nacional de Epidemiología, Prevención y Control de Enfermedades. https://www.dge.gob.pe/portal/docs/vigilancia/boletines/2019/05.pdf.

[2] Ministerio de Salud. Programa presupuestal 0024, Prevención y control del cáncer. https://www.minsa.gob.pe/presupuestales/doc2019/pp/anexo/ANEXO2_6.pdf.

[3] World Health Organization Globocan 2018, Perú. https://gco.iarc.fr/today/data/factsheets/populations/604-peru-fact-sheets.pdf.

[4] Sarria-Bardales G, Limache-García A (2013). Control del cáncer en el Perú: un abordaje integral para un problema de salud pública. Rev Peru Med Exp Salud Publica.

[5] Santos-Ortiz C, Manrique J (2016). Acelerando la innovación en el control del cáncer en el Perú. Rev Peru Med Exp Salud Publica.

[6] Ng D, Ljung B, Bardales R (2016). Developing a breast fine needle aspiration biopsy service in Peru. Ann Global Health.

[7] Abad-Licham M, Galvez Olortegui J, Astigueta J (2018). Diagnostic validity of fine-needle capillary cytology in palpable tumours at the Oncology Institute of Peru. Ecancermedicalscience.

[8] Maita Y, Manrique J, Díaz V (2018). Rol de la biopsia por aspiración con aguja fina (BAAF) en el abordaje diagnóstico de tumoraciones mamarias palpables en el Instituto Nacional de Enfermedades Neoplásicas. Horiz Med.

[9] Cruzado-Sanchez D, Sanchez-Ortiz J, Peralta C (2019). Metástasis de la órbita diagnosticada por biopsia aspiración con aguja fina guiada por ultrasonido: reporte de caso de sitio primario desconocido. Archivos de la Sociedad Española de Oftalmología.

[10] Wu Z, McGoogan JM (2020). Characteristics of and important lessons from the coronavirus disease 2019 (COVID-19) outbreak in China: summary of a report of 72 314 cases from the Chinese Center for Disease Control and Prevention. JAMA.

[11] World Health Organization WHO COVID-19 Preparedness and Response Progress Report February to June 2020. https://www.who.int/publications/i/item/strategic-preparedness-and-response-plan-for-the-new-coronavirus.

[12] Chen CC, Chi CY (2020). Biosafety in the preparation and processing of cytology specimens with potential coronavirus (COVID-19) infection: perspectives from Taiwan. Cancer Cytopathol.

[13] Pambucian S (2020). The COVID-19 Pandemic implication for the cytology laboratory. J Am Soc Cytopathol.

[14] Kashi Z, Torabizadeh Z, Akha O (2011). Combination of aspiration and non-aspiration fine needle biopsy for cytological diagnosis of thyroid nodules. Caspian J Intern Med.

[15] The Royal College of Pathologists Tissue pathways for the diagnostic cytopathology. October 2019. https://www.rcpath.org/uploads/assets/b328ab3d-f574-40f1-8717c32ccfc4f7d8/G086-Tissue-pathways-for-diagnostic-cytopathology.pdf.

[16] Diamantis A, Magiorkinis E, Koutselini H (2009). Fine-needle aspiration (FNA) biopsy: historical aspects. Folia Histochem Cytobiol.

[17] Somocurcio J (2010). Biopsia Punción Aspiración con aguja fina para el diagnóstico de cáncer de tiroides (Unidad de Tiroides del Hospital Edgardo Rebagliati Martins en el periodo del 01 de enero del 2001 al 31 de diciembre del 2005). https://cybertesis.unmsm.edu.pe/handle/20.500.12672/2047.

[18] Colmenero I, Gonzales-Mediero I (2008). Punción aspirativa con aguja fina: utilidad e indicaciones. An Pediatr Contin.

[19] Bardales RH (2014). The Invasive Cytopathologist.

[20] World Health Organization (2020). WHO Coronavirus Disease (COVID-19) Dashboard. https://covid19.who.int/.

[21] Ministerio de Salud del Perú Sala situacional COVID-19. https://covid19.minsa.gob.pe/sala_situacional.asp.

[22] Vigliar E, Laccarino A, Bruzzese D (2020). Cytology in the time of coronavirus disease (covid-19): an Italian perspective. J Clin Pathol.

[23] Srinivasan R, Gupta P, Rekhi B (2020). Indian academy of cytologists national guidelines for cytopathology laboratories for handling suspected and positive COVID-19(SARS-CoV-2) patient samples. J Cytol.

[24] Asociación Peruana de Patólogos Recomendaciones para el manejo y procesamiento de muestras y necropsias en anatomía patológica ante la pandemia COVID 19. vs 01. 2020,12-17.

[25] College of American Pathology (2020). Cytopathology Laboratory Considerations During the COVID-19 Pandemic: College of American Pathologists Cytopathology Committee. https://www.cap.org/laboratory-improvement/news-and-updates/cytopathology-laboratory-considerations-during-the-covid-19-pandemic.

[26] Domansky H (2019). Atlas of Fine Needle Aspiration Cytology.

[27] Parraguirre-Martinez S, De Anda-Gonzalez M, Mantilla-Morales A (2020). Guías de manejo en el laboratorio de anatomía patológica de cadáveres y material biológico con diagnóstico o sospecha de COVID-19. Revista Patología latinoamericana.

[28] Henwood AF (2020). Coronavirus disinfection in histopathology. J Histotechnol.

[29] Sociedad Española de Anatomía Patológica (2020). Sociedad Española de Citología. Medidas de seguridad durante la epidemia por COVID 19 en un servicio de Patología. https://www.seap.es/web/guest/actualidad/-/asset_publisher/cE93/content/medidas-de-seguridad-durante-la-epidemia-por-covid-19-en-un-servicio-de-patologia?inheritRedirect=true.

